# Spatiotemporal dynamics in spiking recurrent neural networks using modified-full-FORCE on EEG signals

**DOI:** 10.1038/s41598-022-06573-1

**Published:** 2022-02-21

**Authors:** Georgios Ioannides, Ioannis Kourouklides, Alessandro Astolfi

**Affiliations:** 1 Department of Electrical and Electronic Engineering, Imperial College London, London, SW7 2AZ UK; 2 Department of Electrical Engineering, Computer Engineering and Informatics, Cyprus University of Technology, 33 Saripolou Street, 3036 Limassol, Cyprus

**Keywords:** Computational neuroscience, Mathematics and computing

## Abstract

Methods on modelling the human brain as a Complex System have increased remarkably in the literature as researchers seek to understand the underlying foundations behind cognition, behaviour, and perception. Computational methods, especially Graph Theory-based methods, have recently contributed significantly in understanding the wiring connectivity of the brain, modelling it as a set of nodes connected by edges. Therefore, the brain’s spatiotemporal dynamics can be holistically studied by considering a network, which consists of many neurons, represented by nodes. Various models have been proposed for modelling such neurons. A recently proposed method in training such networks, called full-Force, produces networks that perform tasks with fewer neurons and greater noise robustness than previous least-squares approaches (i.e. FORCE method). In this paper, the first direct applicability of a variant of the full-Force method to biologically-motivated Spiking RNNs (SRNNs) is demonstrated. The SRNN is a graph consisting of modules. Each module is modelled as a Small-World Network (SWN), which is a specific type of a biologically-plausible graph. So, the first direct applicability of a variant of the full-Force method to modular SWNs is demonstrated, evaluated through regression and information theoretic metrics. For the first time, the aforementioned method is applied to spiking neuron models and trained on various real-life Electroencephalography (EEG) signals. To the best of the authors’ knowledge, all the contributions of this paper are novel. Results show that trained SRNNs match EEG signals almost perfectly, while network dynamics can mimic the target dynamics. This demonstrates that the holistic setup of the network model and the neuron model which are both more biologically plausible than previous work, can be tuned into real biological signal dynamics.

## Introduction

No matter how surprising this might sound, after more than a century of Neuroscience research, researchers still do not comprehend many of the fundamental principles by which the brain controls bodily movements, stores episodic memories and makes plans. The understanding is even more limited when it comes to how the combined activity of millions of neurons in the brain leads to *Consciousness*^1–4^. It is commonly accepted that the human brain is the most Complex System in the whole universe and it is this *Complexity* that prevents unlocking the secrets of its underpinnings. State-of-the-art approaches of tackling these mysteries are based on a powerful computational model: Spiking Recurrent Neural Network (SRNN). The main reason that SRNNs are used is because they are ideal for modelling systems that exhibit *nonlinear* and even *chaotic* behaviour^5^.

In general, Artificial Neural Networks are bio-inspired computational models which have demonstrated tremendous capabilities in various other fields, such as Machine Learning, Robotics, Computer Vision, Natural Language Processing and Control Engineering, mainly due the fact that they can capture *nonlinearities*. In the field of Computational Neuroscience, the dominant techniques in constructing a Cortical Brain Model (CBM) are top-down^4,6–14^ and can typically be thought of as a combination of a model at the neuron-level and a model at the network-level.

At the *neuron-level*, the SRNN is trained to match a target signal using least-squares approaches, such as *FORCE*^4^. The target signal can either be a real-life brain signal, or a simulation of it, which resembles a specific task. Training approaches allows enforcing a certain behavior or dynamics onto the SRNN. This training method is used because (a) it is agnostic toward both the underlying network and the tasks that the network has to solve and (b) the task does not have to be specified in terms of closed-form differential equations in contrast to competing methods, such as Spike-based Coding^9–11^ and the Neural Engineering Framework^12,13^. Also, FORCE takes as input a high dimensional *reservoir*, i.e. a Dynamical System that follows a State-Space Trajectory according to perturbations caused by sequential spatiotemporal data. It then utilizes the complex dynamics of this system to perform computations^4,15–26^. Reservoir Computing is motivated by Cortical Neural Networks and *Online Learning* (i.e. learning in real-time and once the data has been used for training, it is no longer required). All Recurrent Neural Networks (RNNs) are considered to be Dynamical Systems and in particular, SRNNs are a prime example of a reservoir.

At the *network-level*, Clopath et al.^27^, proposed the use of an adjacency matrix (i.e. the matrix which portrays the connectivity and synaptic strengths between neurons) that is a sparse matrix with no additional constraints that are biologically motivated. They also used a simulated target signal of a simplistic sinusoidal waveform, which represents a single musical note and stands for bird sounds. Although not mentioned in the paper, in their open-source code, they have also allowed for “self-connected” neurons. More recently in^28^, Synapse Time Dependent Plasticity (STDP) has been used as a mean of converging synaptic strengths between connected neurons. While this makes their approach more biologically-plausible than previous attempts^27^, there is no formal metric being mentioned assessing this biological plausibility (e.g. through the Small-Worldness Index or the dynamical complexity that aims to measure the consciousness^29^). In addition, all of their excitatory neurons follow adaptive exponential integrate-and-fire dynamics and all their inhibitory neurons follow leaky integrate-and-fire dynamics. As a result, the model at the neuron-level cannot exhibit discontinuities demonstrated in real neuronal activity in the cortex of the mammalian brain. This can be modelled by spike-based neuron models (e.g. Izhikevich neuron model). Furthermore in^30^, Hindmarsh–Rose neurons are utilised which again limits the biological plausibility of the neuronal activity dynamics since no discontinuities can be accommodated. Therefore, albeit this being the state-of-the-art methodology, the biological plausibility of their results remains very limited. Moreover, the *full-FORCE* method^3^ has recently been proposed as an improved method, since it produces networks that perform tasks with fewer neurons and greater noise robustness than FORCE due to the fact that it exploits the full recurrent connectivity by introducing a second network during training. Since biological plausibility remains the key to constructing a CBM, there appear to be a lot of areas of improvement in order to propose a framework which is more realistic and more biologically plausible.

This paper includes the first direct application of the full-FORCE method to spiking neuron models with axonal conductance delays over modular Small-World Networks (SWNs). This is demonstrated in order to explicitly enforce biological plausibility to the CBM. In addition, the aforementioned method applied to spiking neuron models is trained on various real-life Electroencephalography (EEG) signals for the first time. Furthermore, a CBM is proposed which is a trade-off between high biological plausibility, measured by the metric of dynamical complexity (which resembles the Consciousness of the brain) and low regression error between the target and the modelled EEG (time-series) signal. Moreover, a new method is proposed, termed as the *modified-full-FORCE*, which is more biologically plausible than the original full-FORCE method. It is commonly believed that connections between human neurons are directed due to the nature of chemical synapses that connect them. Nonetheless, in^31^, it is described how the directionality between connected neurons in the brain is still unclear and cannot be confirmed. This is because of the limitations of existing noninvasive imaging techniques. Therefore, experiments were repeated for both cases when the connections between neurons are randomly set but are either *directed* or *undirected*. This **highlights** the impact of having directed or undirected synapses on the RMSE of the modelled EEG signals and dynamical complexity of the SRNN. Finally, a new MATLAB toolbox, called “SRNN Brain Modelling Toolbox”, was developed and released open-source on GitHub that can take any kind of time-series signal (e.g. EEG) and reproduce all of the results of this paper.

## Results

### Network topology

**Figure 1:**
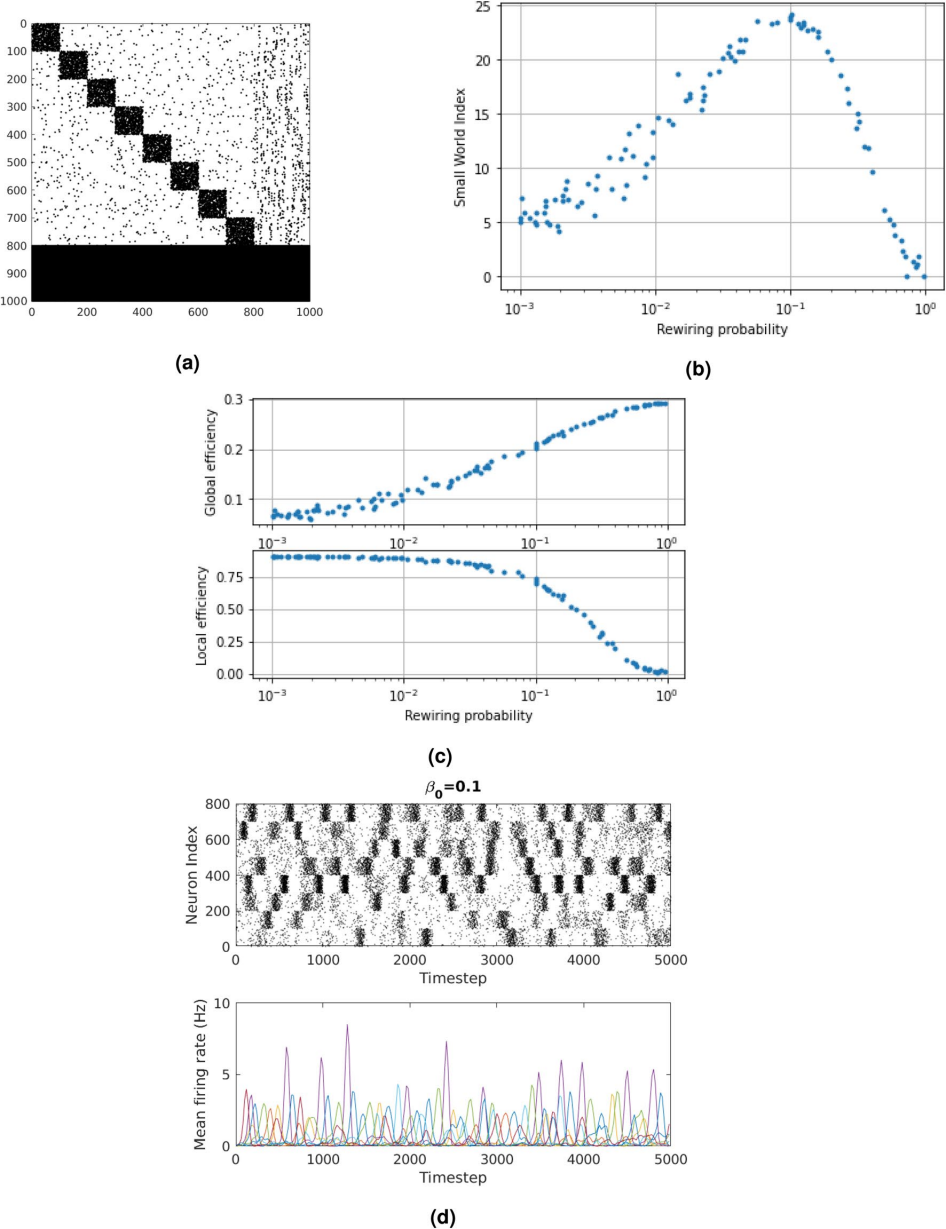
(**a**) Modular SWN with rewiring probability of 0.1 (black dot shows connection between neuron indexes). (**b**) Blue dots show varying rewiring probability and impact on the SWI. (**c**) Blue dots show varying rewiring probability and impact on the GE (top) and the LE (bottom). (**d**) Modular SWN MFR. Black dot shows fired neuron. Different colours show MFR of different modules. These will be referred as the *raster plot*.

The newly proposed framework’s adjacency matrix (Fig. S9 in the Supplementary Material) consists of both inhibitory and excitatory type of cortical neurons using the Izhikevich neuron model^32^. Specifically, in Fig. 1a, indexes 1–800 are excitatory neurons and 801–1000 are inhibitory.

In general, a network is more biologically plausible if it satisfies at least two properties: (a) the adjacency matrix of the generated network forms patterns similar to that of the anatomical brain network (i.e. connectome) as observed by experiments on neurons of a human cerebral cortex (i.e. modular patterns)^33,34^ and (b) each module has a relatively high small-world index^33^. Therefore, as explained in the Methods section and shown in Fig. 1a, the newly proposed framework makes use of $M=8$ modular Small-World Networks (SWNs).

In order to examine whether or not a network does satisfy the small-worldness property, three Graph Theoretic metrics are calculated across a range of intra-cluster rewiring probabilities, $\beta _0$. Namely these are: (1) Small-World Index, (2) Global Efficiency and (3) Local Efficiency. Using a graph with 500 nodes, at the optimal value $\beta _0$ = 0.1, the SWI reaches the peak value of 24 (Fig. 1b) while there is a good trade-off between global efficiency of 0.2 (top of Fig. 1c) and local efficiency of 0.75 (bottom of Fig. 1c), at the expense of one another. These are statistics which characterize the efficiency in which information can be broadcast through a network at the local (i.e. module-level) and global level (i.e. the whole network).

The three metrics were also measured after repeating the same experiments, but varying the total number of nodes (representing neurons). To illustrate the trend of how SWI changes with an increasing number of nodes in the network, SWNs with various other numbers of nodes (20, 200) were generated for the same range of values of $\beta _0$ (Supplementary Figs. S1–S2, respectively). Regarding the modular SWNs with 20 and 200 nodes, very similar results to that of 500 nodes were observed, thus suggesting that a modular SWN with $\beta _0$ = 0.1 is the one with more biological plausibility (since it experiences a much higher SWI) than the other ones, irrespective of the number of nodes.

With both types of rewiring probabilities kept constant ($\beta _0$ = 0.1, $\beta _1$ = 0.1) the adjacency matrix of a modular SWN with 800 excitatory (labelled 1–800) and 200 inhibitory neurons (labelled 801–1000) is constructed (Fig. 1a). Therefore, there are four types of neuron connections (which can be seen by breaking up the adjacency matrix into four constituent matrices): (1) excitatory-to-excitatory, (2) excitatory-to-inhibitory, (3) inhibitory-to-inhibitory and (4) inhibitory-to-excitatory. SWN with $\beta _1$ = 0.1 appears to be the one that resembles connectomes from empirical studies^33,34^ more than the other ones.

These results verify similar computational experiments which were conducted by Shanahan^35^ in the past, but not in the context of neuroimaging signals (either real or simulated) as it is the case here.

### Dynamical complexity

In order to *evaluate* the biological plausibility aspect of the constructed network, the metric of dynamical complexity^29^ (which resembles the Consciousness of the brain) is used. Axonal conductance delays between connected neurons are put in place. These delays refer to the amount of time needed for an action potential to reach from its initiation site near the neuronal soma to the axon terminals so that it can be transmitted to other neurons through synapse connections. It is not realistic if they are not put in place, as it would imply that all neuron are all at the same place, and have the same distances between them.

In Fig. 1d, the Mean Firing Rate (MFR) and raster plot for an SRNN with modular SWN structure and axonal conductance delays are shown. All of the modules are mostly acting independently, but they also have some influence on each other (i.e. collaborative activity). This can be verified by observing the raster plot at time instants when different modules densely fire up at the same time whilst at other time instants, they do not. The dynamical complexity for this network is 0.1122. Moreover, increasing the rewiring probability, $\beta _0$, causes a drop in dynamical complexity as illustrated in Table 1. This is also visually confirmed in Figs. S10–S13 in the Supplementary Material. By increasing the rewiring probability, independent neuronal activity starts decreasing and the modules no longer have independent activity patterns (e.g. see Fig. S13 in the Supplementary Material). Moreover, independent activity can also be verified by observing the MFR of each module. For instance, in Fig. 1d, each different colour in the MFR plot represents the firing frequency of a module. It can be easily seen that there are some overlaps but there is largely independent activity (by observing the peaks of the MFRs). Higher firing frequency implies more neuronal activity.

On the other hand, looking at Fig. S13 in the Supplementary Material, the MFR is approximately equally low across all modules. Thus, it is evident that modular SWNs exhibit higher neuronal activity than randomly connected ones of the same neuron population size. Higher neuronal activity can be interpreted as a more efficient network carrying out complex tasks and exhibiting complex dynamics. Increasing the re-wiring probability has an effect of decreasing the modular SWN structure, and the neuron populations fire with more fully integrated (“interlocked” spiking patterns) approaching a neural complexity of zero. This would imply that the whole neuron population does exactly the same thing which is not biologically plausible.

**Table 1:** Dynamical complexity of the topologies, $p \equiv \beta _0$.

	Dynamical complexity
*p = 0.1, N = 1000*	**0.1122**
*p = 0.2, N = 1000*	0.0625
*p = 0.3, N = 1000*	0.0793
*p = 0.4, N = 1000*	0.1010
*p = 0.5, N = 1000*	0.0798

Significant values are in bold.

### EEG modelling

**Figure 2:**
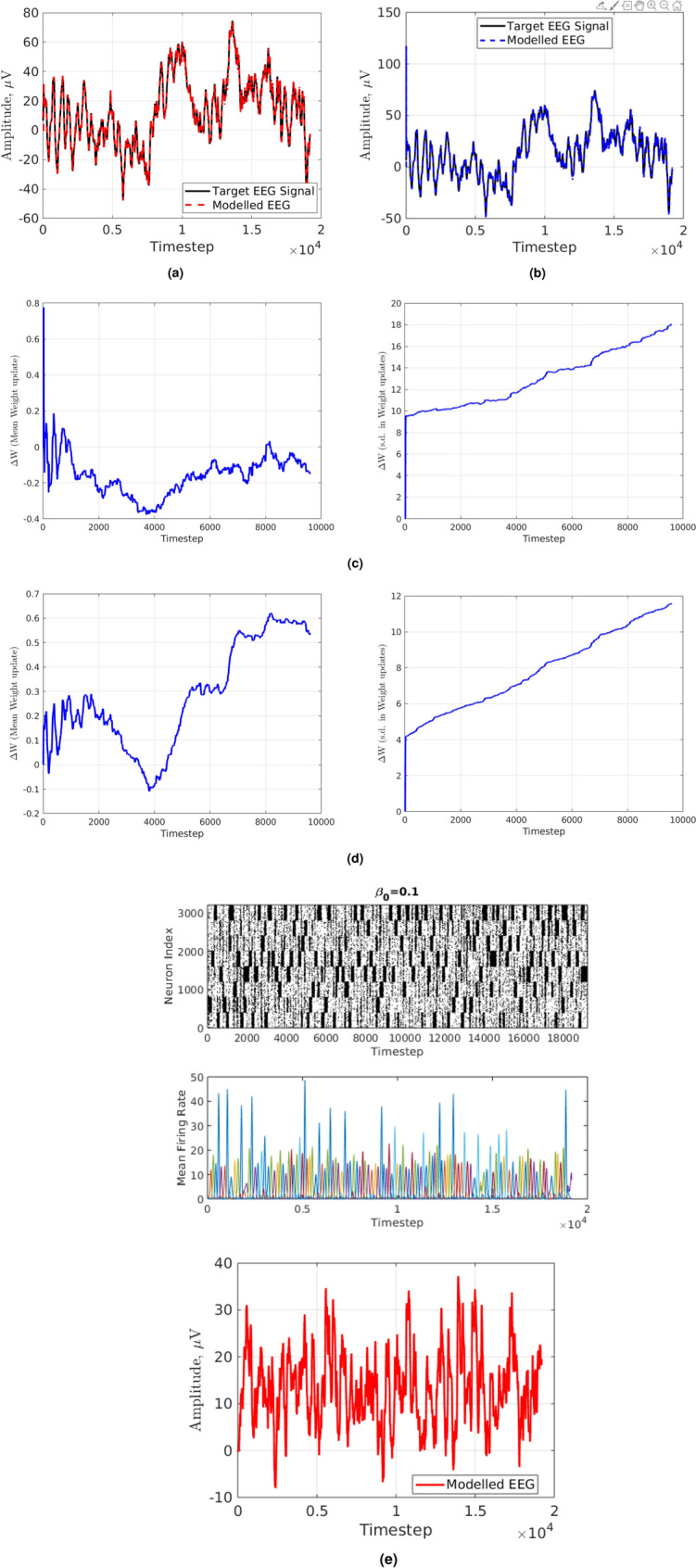
(**a**) Using modified full-FORCE. Black solid line shows the “Target EEG signal”. Red dotted line shows the “Modelled EEG Signal”. (**b**) Using FORCE method as adapted by^27^. Black solid line shows the “Target EEG signal”. Blue dashed line shows the “Modelled EEG Signal”. (**c**) Modified full-FORCE with 1000 neurons. Blue solid line shows mean weight change (left). Blue line shows s.d. weight variation (right). (**d**) Modified full-FORCE with 4000 neurons. Blue solid line shows mean weight change (left). Blue line shows s.d. weight variation (right). (**e**) Modified full-FORCE with 4000 neurons. Raster plot (top). Red Solid line shows “Modelled EEG Signal” (bottom).

To evaluate the performance in modelling the target EEG signals, the metric of Root Mean Squared Error (RMSE) is used. As a result, a multi-objective optimization problem is formulated where the desired objectives are to maximize the dynamical complexity and minimize the RMSE subject to retaining a modular small-world neural network with axonal conductance delays. In Fig. 2a channel 14 of the EEG signals dataset is modelled using *modified full-FORCE* and in Fig. 2b the method used in^27^ is used. The method used in^28^ is not applicable on time-series data such as a biological signal (i.e. EEG).

The RMSE using the modified full-FORCE is *183.3* whilst using the method used in^27^ gives *319.5*. While both methods perform relatively well in respect to the RMSE, the latter fails to maximize dynamical complexity because it does not make use of axonal conductance delays and modular SWN structure (see Fig. S9 in the Supplementary Materials depicting its adjacency matrix which shows no modular SWN structure). It should be noted that SWNs can exhibit short-term memory, which is the ability to hold information between different states in a Dynamical System. Provided that the modified-full-FORCE combined with modular SWN architecture is used, the state-space trajectory of an EEG signal can be repeated, if given the same initial conditions (i.e. initialization of the ionic currents that flow through the network). This is shown in Fig. 2a.

### Modified full-force

One of the novel contributions is a more biologically-plausible learning procedure. A modification was made to the Optimization procedure of the original full-FORCE method introduced in^3^ and the modelled output at every timestep of the process is **not** fed back into the network. The magnitude of ionic current input affects the inter-spike behaviour of neurons. By not having a feedback loop, the ionic currents flowing through the network are not tampered with a non-biologically plausible current flow. Moreover, by not having a feedback loop the neuron population’s dynamics are **not** all tuned with the same feedback term which forces them to spike in **interlocked** patterns causing a **decrease** in dynamical complexity. As a result, the optimization procedure only makes use of the output current of each neuron that has spiked. This newly proposed method is termed *Modified-full-Force*. To visually inspect the difference in neuronal activity and the modelled EEG signal using the original *full-Force* method versus the *Modified-full-Force* refer to Figs. S14 and S15 (original *full-Force*) in the Supplementary Material and the rest of this chapter for *Modified-full-Force*. *Modified-full-Force* is an Online Learning algorithm.

In Fig. 2c, an SRNN’s mean weight updates across channel 14 of the EEG dataset is shown. The SRNN has 800 excitatory neurons, 200 inhibitory neurons and a total of 2000 random connection between them. In Fig. 2d, an SRNN with 3200 excitatory neurons, 800 inhibitory neurons and a total of 8000 random connections is used. It is evident that as increasing the number of neurons (keeping constant ratios of 80% excitatory neurons and 20% inhibitory neurons), **and** the number of random connections between them (i.e. the number of edges on a graph) reduces the RMSE and the standard deviation of weights update tends to decrease, suggesting that the underlying network is a good guess for modelling the EEG signals. Considering that around 30–500 million neurons are responsible for EEG signal recordings^36^, the neuron population number was increased while enforcing biological constraints (i.e. Conductance delays, modular Small-Worldness). Moreover the mean weight updates is around zero suggesting that the linear transformation between neuronal activity and the EEG signal is relatively constant.

Lastly, in Fig. 2e (bottom), it can be shown that the underlying dynamics of an EEG signal can indeed be captured using modified-full-FORCE. In Fig. 2e, the SRNN was freely integrated without applying any optimization for 19,200 time-steps ahead of the target EEG signal illustrated in Fig. 2a. The freely modelled EEG signal shown in Fig. 2e can be thought of as the evolution of the neuronal system activity, provided all variables remain constant (i.e. external stimuli that affect neuronal activity).

### Directed networks

**Figure 3:**
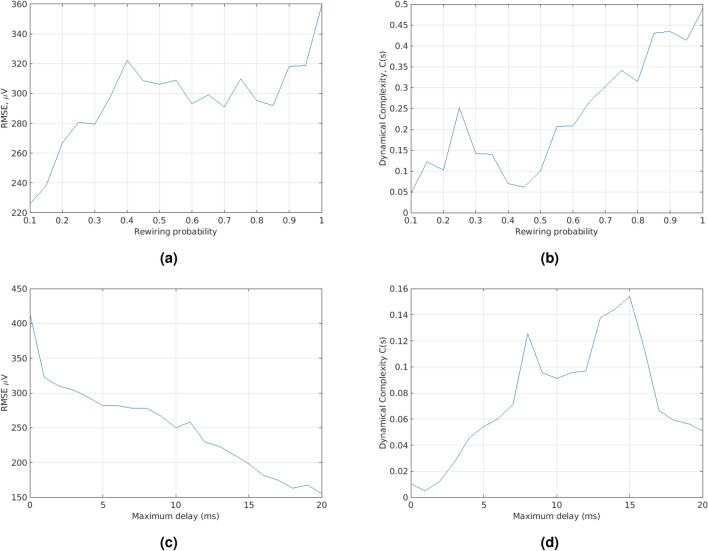
(**a**) Blue solid line shows RMSE with varying re-wiring probability (800 excitatory neurons and 200 inhibitory neurons). (**b**) Blue solid line Dynamical Complexity with varying re-wiring probability (800 excitatory neurons and 200 inhibitory neurons). (**c**) Blue solid line shows varying conductance delay and assessing RMSE. (**d**) Blue solid line shows varying conductance delay and assessing dynamical complexity.

In Fig. 3a, the RMSE with varying rewiring probability is illustrated. Observing Fig. 3a, at lower re-wiring probabilities which are closer to 0.1 and SWI is maximized, a lower RMSE is produced. In general, the EEG signal was successfully matched and hence, it can be concluded that the full-Force *can* be applied to biologically plausible neural networks and it *can* handle well their excessively more complex dynamics than the ones of the networks originally used when full-Force was introduced in^3^.

However, the RMSE plot in Fig. 3a mostly informs about how robust the Optimization algorithm is when applied in biologically-plausible neural networks. In order to quantify how biologically plausible the network is, the dynamical complexity should be accounted of in addition to its small-world modular property. In order to compute the dynamical complexity, first the MFR has to be evaluated for each of the eight modules. The MFR for each module is a time-series which illustrates the MFR per module at every timestep across the integration horizon. The MFR time-series of each module is differenced twice (i.e. approximating its second derivative). Differencing can help stabilize the mean of a time-series and as a result can reduce the trend, a technique known as *detrending*. Given a signal, $y_t$, second order differencing makes the following modification:

$$\begin{aligned} y''_{t}&= y'_{t} - y'_{t - 1} \\&= (y_t - y_{t-1}) - (y_{t-1}-y_{t-2})\\&= y_t - 2y_{t-1} +y_{t-2}. \end{aligned}$$

Thus, Information Theoretic metrics such as MI and Shannon’s Entropy utilized by *C*(*S*) for the estimation of the dynamical complexity can be used more reliably.

In Fig. 3b, the dynamical complexity with varying rewiring probability is illustrated. It is obvious that dynamical complexity, *C*(*S*), is maximized when the rewiring probability is between 0.2 and 0.3. This suggests that the most biologically plausible networks which have a high SWI also have a high dynamical complexity. High dynamical complexity implies a balance of segregated and integrated activity which implies a lot of interaction between modules and at the same time many components of the system are carrying out independent activity. Moreover, higher rewiring probabilities (i.e. $>0.5$) that do not produce modular SWNs have been included in the plot but *cannot* be considered biologically plausible. This serves as an example that a single metric (e.g. dynamical complexity) cannot be used on its own for evaluating biological-plausibility. Instead, it should be combined with graph theoretic metrics such as the SWI to get a more reliable interpretation.

To pinpoint the best configuration that retains biological plausibility and has a relatively low RMSE, the number of directed connections, which affects the degree distribution of the network, and the number of neurons was varied in Table 2. This was conducted in order to observe how these two parameters affect both the dynamical complexity and RMSE, while retaining modular small-worldness (i.e. rewiring probability approximately 0.1–0.2) and maximum conductance delay of 20 ms.

**Table 2:** Biological plausibility assessment.

	N	RMSE	Dynamical complexity	No. of connections
p = 0.1	2000	166.8	0.09166	2000
p = 0.1	3000	122.9	0.02131	3000
p = 0.1	4000	117.1	0.02750	3500
p = 0.2	2000	162.4	0.06358	2000
p = 0.2	3000	129.3	0.03713	3000
p = 0.2	4000	107.8	0.04118	3500

Observing Table 2, it is obvious that as the number of neurons, *N*, is increased, the RMSE decreases, however, this is not also true for the dynamical complexity. This suggests that a better method is needed for tuning between the two. However, there is trend that as the rewiring probability increases (even when the number of neurons was also increased), dynamical complexity mostly follows a decreasing pattern. The reason that RMSE decreases as the number of neurons is increased and dynamical complexity is decreased, is because most of the neurons (irrespective of module) start doing the same type of activity. Thus, it is easier for the Optimization procedure to optimize since all neurons have correlated activity patterns. Hence, using the correlation matrix in the RLS procedure better optima can be found. Therefore, RMSE is decreased at the expense of losing biological plausibility. Varying the maximum conductance delay between neurons also has an effect in the dynamical complexity and RMSE. In essence, varying the conductance delays allows for modelling short and long non-myelinated central axons. When the maximum conductance delay is kept low, the whole system has more beneficial properties in modelling speed-sensitive processes such as reflexes, perceptual skills and escape responses. Moreover, fast axons occupy around $4 \times 10^{4}$ times more volume in the brain than slow axons. On the other hand having a high maximum conductance delay allows for slower reactions which could for example be exhibited when a person is under the influence of drugs such as antidepressants. In Fig. 3c, the variation of the maximum conductance delay between neurons is illustrated.

Having a very low maximum conductance delay implies that the activity and spiking patterns of the neurons change rapidly. As a result, this makes it harder to optimize for. Increasing the maximum conductance delay allows the neuron population to also exhibit slower changes in spiking patterns. The reason is that excited neurons take longer to excite other neurons that are connected to them through synapses. Therefore, this makes it easier for the optimization procedure to optimize for. In Fig. 3d, the impact of varying the maximum conductance delay on the dynamical complexity of the neuron population is illustrated.

**Figure 4:**
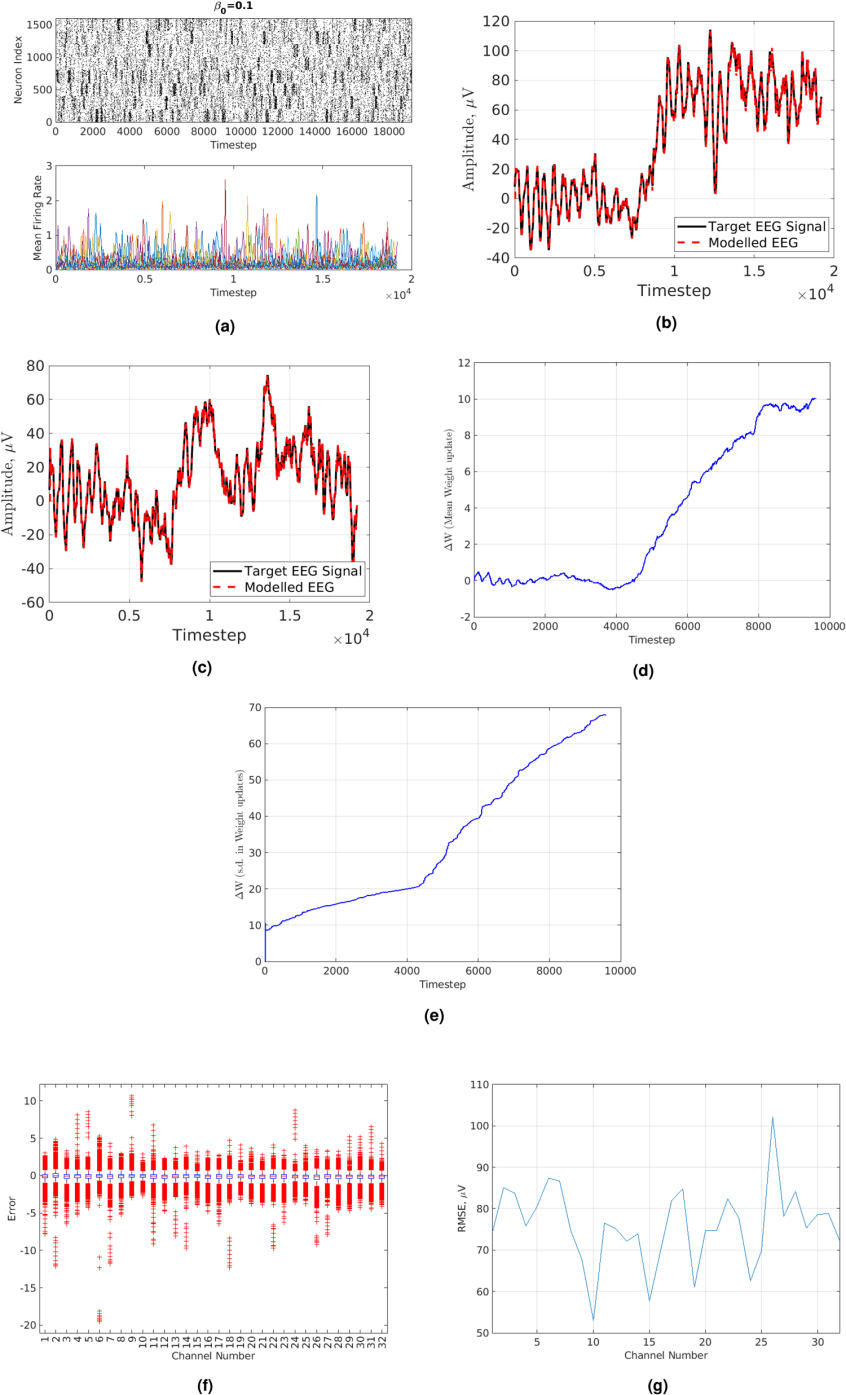
(**a**) Directed topology. Neuronal activity and MFR (raster plot). (**b**) RMSE of directed network for channel 26 using 15 ms conductance delay. Red dashed line shows “Modelled EEG signal”. Black solid line shows “Target EEG signal”. (**c**) Directed biologically-plausible SRNN with max. conductance delay of 15 ms-channel 14. Red dashed line shows “Modelled EEG signal”. Black solid line shows “Target EEG signal”. (**d**) Blue solid line shows Mean Weights update using Directed biologically-plausible SRNN. (**e**) Blue solid line shows s.d. of Weights update using Directed biologically-plausible SRNN. (**f**) Boxplots of error for 32 EEG channels. Red crosses are data points beyond the whiskers. (**g**) Black solid line shows RMSE of directed network for 32 EEG channels.

Having a relatively very low maximum conductance delay implies that neurons are all exhibiting rapid spiking changes. However, these changes happen to all of the neurons at the same time and hence are exhibiting the same type of activity. The most probable reason that the Optimization procedure does not perform as well as when the maximum conductance delay is higher, is because even though these changes happen at a global level (not just modular), they take place too rapidly. Using the optimal pair of values in Table 2 (i.e. the first row), Fig. 4 was generated. A maximum conductance delay of 15 ms, Channel 14 (i.e. Cz electrode) was modelled in Fig. 4c.

The RMSE in Fig. 4c was *73.9*. In Fig. 4a, the network topology used for modelling the EEG signal is illustrated. In Fig. 4d,e the mean and standard deviation of the optimal weights matrix updates per Optimization step are shown.

It is obvious that the standard deviation is much higher than in the optimal weights matrix updates in previous sections. This is because of the dynamical complexity of the network being much higher than before due to more biologic plausibility as well as due to the $\alpha$ parameter of the Tikhonov regularization matrix being set too high (i.e. 2200). This was not possible before due to the feedback that existed in the original full-Force method. In Fig. 4f, the boxplot of errors during the Replay of State-Space Trajectory Phase has been plotted. Observing Fig. 4f, it can be seen that across all channels of the EEG dataset, the average value of the error is approximately zero. In Fig. 4g, the RMSE for each EEG channel in the dataset is shown.

It is evident that across all 32 channels the RMSE was similar with the exception of channel 26. For this reason, the modelled and original EEG of channel 26 are shown in Fig. 4b. The RMSE for the modelled EEG of channel 26 was *102.2*. Even though it exhibited the highest RMSE out of all channels it was modelled relatively well since the regression error was relatively low.

### Undirected networks

**Figure 5:**
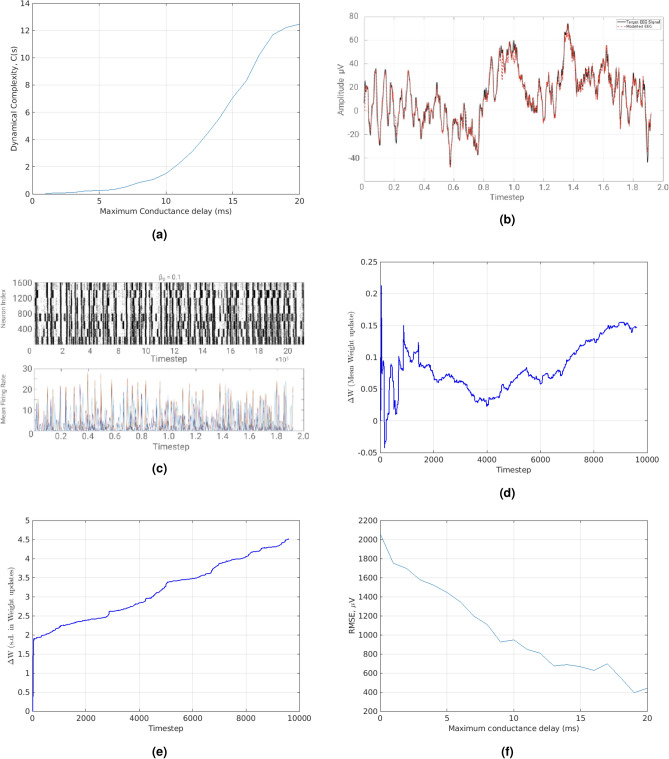
(**a**) Blue solid line shows Dynamical Complexity of undirected network. (**b**) Undirected biologically-plausible SRNN. Red dashed line shows “Modelled EEG signal”. Black solid line shows “Target EEG signal”. (**c**) Undirected topology Neuronal activity and MFR (raster plot) (**d**) Blue solid line shows Mean Weights update using biologically-plausible SRNN. (**e**) Blue solid line shows s.d. of Weights update using biologically-plausible SRNN. (**f**) Blue solid line shows RMSE of undirected network for varying maximum conductance delay.

Undirected SRNNs with the extended biological plausibility attributes introduced in this section are very computationally intensive to model and optimize. Taking into consideration the best configuration illustrated in Table 2, the best maximum conductance delay, which gives best dynamical complexity, is derived using an undirected version of the directed network. In Fig. 5a, the dynamical complexity of the undirected network is illustrated against varying maximum conductance delay.

As the maximum level of conductance delay is allowed to be higher, the dynamical complexity of the undirected network increases. The best dynamical complexity is when the maximum conductance delay is set to 20 ms. However, having a maximum conductance delay greater than 15 ms makes it much more computationally expensive for EEG signal modelling. In Table 3, the performance results of the Undirected SRNN are shown. The $\alpha$ parameter of the Tikhonov matrix was set to 2. Empirically, it was noticed that undirected networks require $\alpha<< N$, otherwise divergence occurs. This was not true for directed networks.

**Table 3:** Undirected SRNN.

	N	RMSE	Dynamical complexity	No. of undirected connections	Max. delay
p = 0.1	2000	691.8	7.6386	2000	15 ms

The undirected network exhibits worse performance (i.e. higher RMSE) than the directed network, because it has more complicated dynamics that are harder to account for. However, the more complicated dynamics produced a **much** higher dynamical complexity rather the directed network. In order to qualitatively show the performance of the modified-full-Force of the undirected network, the modelled EEG of channel 14 (i.e. $\texttt {Cz}$ electrode) is illustrated in Fig. 5b.

By observing Fig. 5b, it can be deduced that the main source of the errors stems from high frequency oscillations. Nonetheless, as shown in previous sections, increasing the number of neurons and at the same time keeping a ratio of neurons that favours dominance towards the Regular Spiking and Intrinsically Bursting types rather the Chattering type allows for monotonically decreasing RMSE. In Fig. 5c, the raster plot of excitatory neuronal activity and MFR of the undirected network is illustrated.

The MFR of the undirected network clearly shows extended independent and collaborative activity between different small-world modules than the directed one. This can be seen from the large peaks of several modules intersecting with smaller peaks from other modules. This shows there is a lot of firing activity developing in a module while at the same time a lower amount of firing activity going on in other modules. This is the prime reason for the large increase in dynamical complexity compared to the directed network. Moreover, the MFR in the undirected network was much higher than the MFR in Fig. 1d due to the much higher dynamical complexity that exhibits. Figure 5d,e illustrate the mean and standard deviation of the optimal weights using the undirected network. It is clear that the weight updates are frequently changed however, due to having a much lower $\alpha$ parameter, the standard deviation of the optimal weights matrix updates is lower than it was in the directed network.

In Fig. 5f the RMSE is plotted against varying maximum conductance delays. As the dynamical complexity increases, the RMSE decreases. This is due to the same reasons outlined above for directed networks.

**Figure 6:**
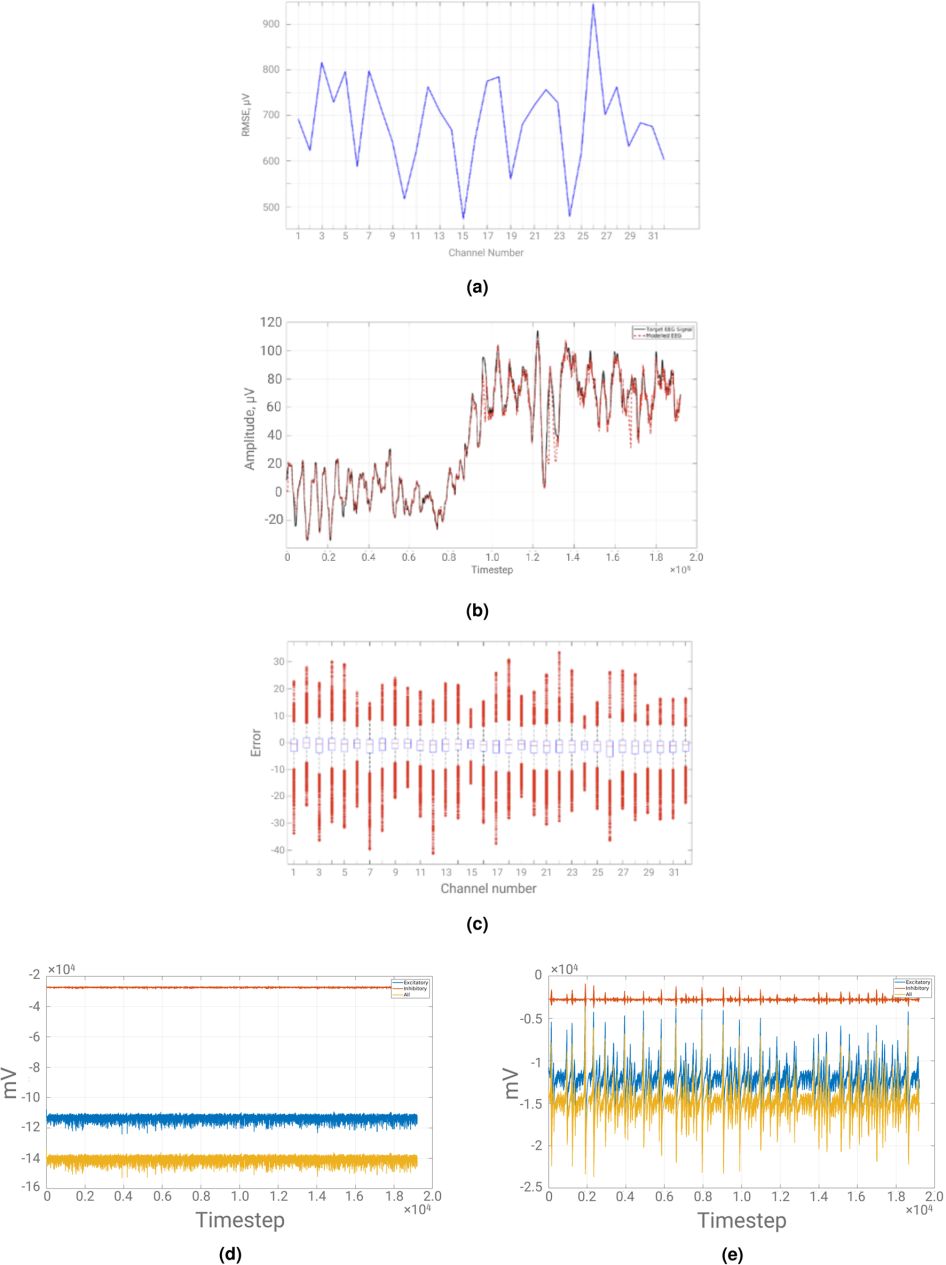
(**a**) Blue solid line shows the RMSE of undirected network for 32 EEG channels. (**b**) Undirected biologically-plausible RSNN with max. conductance delay of 20 ms-channel 26. Red dashed line shows “Modelled EEG signal”. Black solid line shows “Target EEG signal”. (**c**) Undirected network Boxplots of error for 32 EEG channels. Red crosses are data points beyond the whiskers. (**d**) Directed Network. Orange solid line shows voltage (mV) variation of inhibitory neurons. Blue solid line shows voltage (mV) variation of excitatory neurons. Yellow solid line shows voltage (mV) variation of the collective (i.e. sum) voltage (mV) of excitatory and inhibitory neurons. (**e**) Undirected Network. Orange solid line shows voltage (mV) variation of inhibitory neurons. Blue solid line shows voltage (mV) variation of excitatory neurons. Yellow solid line shows voltage (mV) variation of the collective (i.e. sum) voltage (mV) of excitatory and inhibitory neurons.

From an Optimization point of view, the RMSE has approximately the same trend across channels (see Fig. 6a) as for the directed network case. However, the RMSE is higher in magnitude than the directed network case. The RMSE in Fig. 6b was *796.9*. This is approximately 200 $\upmu$V less than when the maximum conductance delay was 15 ms.

In Fig. 6c, the boxplots of the errors of the Undirected network with maximum conductance delay of 15 ms are shown. While the error is approximately zero across all EEG channels, there are many more outliers than there were in the case of directed network, thus portraying its elevated dynamical complexity.

### Comparison of directed and undirected networks

Both types of networks exhibit **similar patterns** in terms of RMSE accross the various EEG channels. Directed networks could be trained to have a lower RMSE than Undirected networks, but also have a lower dynamical complexity than Undirected networks. While the directionality of the human connectome is still an open question, the impact of full directionality against full undirectionality has been investigated from both the regression perspective and the biological plausibility point of view, as determined by the RMSE and the dynamical complexity respectively. *Directionality* in synapse connections vastly affects the dynamical complexity of the networks and the value of RMSE between the modelled and the target EEG signals. In Fig. 6d,e, the collective voltage behaviour of the directed network and undirected network are shown, respectively.

In the Undirected Network case, the neural spikes are higher than the mean voltage value of the specific neuron type. On the other hand, in the Directed network case, the voltage spikes are approximately equal to the mean voltage value of each neuron type. Therefore, an important research question that needs to be addressed next is whether the connectome is either *fully directed* or *fully undirected* or a *mixture of the two*. This could unlock further secrets of the brain and suggest the path for next research steps on the computational side of Neuroscience.

## Methods

### Network topology

In order to construct a network topology that is small-world (so that it can be more biologically plausible than the state-of-the-art) with no self-loops (since real-life neurons cannot be connected to themselves), a random network, $\mathscr {G}$, using the Watts–Strogatz model and a fixed intra-cluster rewiring probability, $\beta _0$, was generated, as explained in the Supplementary Material. Motivated by the ‘Six Degrees of Separation’, a known phenomenon in social networks^37^ and frequently associated with the Milgram experiment^38^, the neighbourhood size per node on the graph, *K*, was set to 6, which produces networks with relatively high SWIs. Then, while keeping *K* constant, the values of the SWI, $\sigma (\mathscr {G})$, local efficiency, $E_L(\mathscr {G})$, and global efficiency, $E_G(\mathscr {G})$, were measured in a series of experiments generating multiple networks, while the intra-cluster rewiring probability $\beta _0$ was varied. Due to the stochastic nature of the generated networks, the arithmetic mean for each of the three Graph Theoretic metrics was recorded over 100 trials with the same $\beta _0$. The total number of nodes, $N_m$, was set to 100.

### Small world network (Watts–Strogatz model)

An SWN is a particular type of graph whose typical (i.e. expected value) distance, *L*, between any two randomly nodes grows logarithmically with network size, *N* (i.e. number of nodes). In other words:

1 $$\begin{aligned} L \propto \log N \end{aligned}$$

A particular category of SWN is the *Watts-Strogatz model*, which is a random graph that exhibits *small-world properties*. A graph has small-world properties if it has a *high* clustering coefficient and a *small* average path length.

The steps for generating an SWN are provided in Algorithm 1, where *N* is the number of nodes, *K* is the mean degree and $\beta$ is the rewiring probability. The model satisfies $0\le \beta \le 1$ and $N\gg K\gg \ln N\gg 1$, and creates an undirected graph with *N* nodes and $\frac{NK}{2}$ edges.

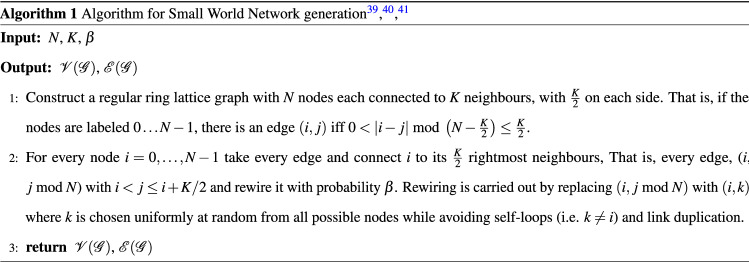



A *lattice graph* is a graph that is part of a euclidean space $\mathscr {R}^N$ that forms a regular tiling. A regular tiling is a plane with one or more non-overlapping and no-gapped geometric shapes. Algorithm 1, produces approximately $\beta {\frac{NK}{2}}$ non-latticed edges. When $\beta$ (i.e. the rewiring probability) is varied the number of non-lattice edges changes. In the extreme case when $\beta = 0$, a *regular ring lattice* graph is formed.

By increasing $\beta$, the randomness in the graph is increased, and in the extreme case when $\beta = 1$, the Watts–Strogatz model closely resembles a purely random graph (i.e. $p=1$). A *purely random graph*, built according to the Erdos–Renyi model^42^, exhibits a *small* average path length along with a *small* clustering coefficient. An SWN can be observed at $\beta = 0.1$ (i.e. $p=0.1$), which is somewhere in-between a regular ring lattice and a purely random graph.

### Small-Worldness Index

The Small-Worldness Index (SWI), $\sigma$, can be computed through direct comparison of a network’s clustering coefficient and average path length to a (purely) random graph which has on average, the same degree distribution. The mean path length, $\lambda _{\text {rand}}$, of a random graph which is made up of *N* nodes and average degree distribution, *K*, is on average $\frac{ln(N)}{ln(K)}$ and its corresponding clustering coefficient, $\gamma _{\text {rand}}$, is on average $\frac{K}{N}$. Considering a network, $\mathscr {G} = (\mathscr {V}, \mathscr {E})$, which is composed of *N* nodes and has average degree distribution *K*, then it is an SWN if:

1. It is a sparse graph (i.e. $K<< N$)
2. Its clustering coefficient is higher than the random graph’s
3. It has an average path length which is similar to the average path length of a random graph.

The SWI, $\sigma _G$, can be quantified by comparing the ratio of the clustering and the path length of a given network, *G* to an equivalent random network with same degree on average:

2 $$\begin{aligned} \sigma _{G} = \frac{\gamma _{G}/\gamma _{\text {rand}}}{\lambda _{G}/\lambda _{\text {rand}}} \end{aligned}$$

### Global and local efficiency

In^43^, Latora and Marchiori introduced a statistic which characterizes the efficiency that information can be broadcasted through a network (i.e. a graph). Consider a network $\mathscr {G} = (\mathscr {V}, \mathscr {E})$ with *N* nodes. The efficiency between any two nodes in $\mathscr {G}$ can be defined as $\frac{1}{\lambda }$, where $\lambda$ denotes the path length from node $\texttt {i}$ to node $\texttt {j}$ in the network. In closed form:

3 $$\begin{aligned} \text {Efficiency}(\texttt {i,j}) = \frac{1}{\lambda _{\texttt {i,j}}} \end{aligned}$$

Using this metric, the information broadcast efficiency can be quantified with a maximum value being 1 in the case were nodes $\texttt {i}$ and $\texttt {j}$ are neighbours directly connected together. Therefore, *global efficiency* of a network $\mathscr {G}$ is defined as:

4 $$\begin{aligned} E_G(\mathscr {G}) = \frac{1}{N(N -1)}\sum _{\texttt {i} \ne \texttt {j}} \frac{1}{\lambda _{\texttt {i,j}}} \end{aligned}$$

Given that in small-world networks communities exist, it is also useful to define the efficiency over a neighbourhood around a node. Formally, let $\mathscr {G'} = (\mathscr {V'}, \mathscr {E'})$ be a sub-network of $\mathscr {G}$ such that $\mathscr {V'} \subseteq \mathscr {V}$ describe all the neighbours of node $\texttt {i}$. Moreover, the set $\mathscr {E'} \subseteq \mathscr {E}$ describes the set of edges that join the nodes in $\mathscr {V'}$. Then, the *local efficiency* of a network $\mathscr {G}$ is described by:

5 $$\begin{aligned} E_L(\mathscr {G}) = \frac{1}{N} \sum _{i \in G} E_G(\mathscr {G}_{i}) \end{aligned}$$

Finally, if no path exists between any two nodes $\texttt {i,j}$ where $\texttt {i} \ne \texttt {j}$, then the Efficiency($\texttt {i,j}$) between these two nodes is 0.

Apart from being small-world the network should also be modular, since not all small-world networks are necessarily modular. So, using the optimal value of $\beta _0 = 0.1$ that produces the highest SWI, a small-world modular network, which consists of M = 8 modules of $N_m$ = 100 excitatory neurons each (so a total of $N_e = M \times N_m$ = 800) and a single random graph module of $N_i$ = 200 inhibitory neurons was constructed. The excitatory modules were randomly connected between each other using the inter-cluster rewiring probability, $\beta _1$, which was also set to 0.1. These modules are sparsely randomly connected between each other to model the long-range connections between different cortex areas in the human brain. In general, the network topology is typically defined by an adjacency matrix, $J \in R^{N \times N}$, whose elements indicate whether the node *i* (representing a neuron) is connected through an edge to a node *j*. If the node *i* and the node *j* are not connected, then the entry (*i*, *j*) is equal to zero. Otherwise, it is equal to the synaptic strength between the two nodes. In the special case of a fully undirected graph, the adjacency matrix is a symmetric. It should be noted that in^31^, it is described how the directionality between connected neurons in the brain is still unclear and cannot currently be confirmed. This is because of the lack of noninvasive neuroimaging techniques. Therefore, the adjacency matrix of an undirected network with $N = N_e + N_i = 1000$ nodes was plotted.

The purpose for which all of the above experiments were conducted was to explore the Network Science side of the constructed network $\sigma (\mathscr {G})$, without using any dataset. The same experiments were also repeated for the static weight matrix of the NSN.

### Dynamical complexity

In order to explore the *biological plausibility* side of the constructed network, the metric of dynamical complexity (which resembles the *Consciousness* of the brain) is utilised. The Neural Complexity is a measure which is equal to Shannon’s Entropy, *H*(*S*), a well-known metric in *Information Theory*. It measures how much information is carried in the variable *S* over time. When the variables within *S* are real-valued, then *H*(*S*) can be computed as follows:

6 $$\begin{aligned} H(S) = \frac{1}{2}\ln ({(2\pi e)^{N}\mid COV(S) \mid }) \end{aligned}$$

where *COV*(*S*) denotes the covariance matrix of *S*.

The integration of the system, *I*(*S*) can be computed as follows:

7 $$\begin{aligned} I(S) = \sum _{i=1}^{n} H(X_i)-H(S) \end{aligned}$$

The Mutual Information (MI) of a system can be computed as follows:

8 $$\begin{aligned} MI(X;S-\{X\}) = H(X) + H(S-\{X\})-H(S) \end{aligned}$$

MI measures how much information there is in one part of the system that can explain another. In the case of a neural system, the objective is to evaluate the degree of influence that each component of the system, *X*, has on the whole system, *S*.

In^29^, Tononi et al. introduced a metric to quantify segregation and integration of activity in a system. It was described that a system which exhibits a balanced amount of segregated and integrated activity is capable of a large repertoire with multiple spectrum of responses. Moreover, it has an efficient utilization of its resources to provide a merged response from all of its components to an induced stimulus. The dynamical complexity, *C*(*S*), is given by:

9 $$\begin{aligned} C(S) = \sum _{i=1}^{n} MI(X_i;S-\{X_i\})-I(S) \end{aligned}$$

The objective is to maximize the dynamical complexity:

10 $$\begin{aligned} \max C(S) \end{aligned}$$

To explore the biological plausibility of the network, a set of 1000 Izhikevich neurons (800 excitatory and 200 inhibitatory) were generated and simulated for a total of 5 seconds (represented as 5000 timesteps of 1 ms each) with varying values of rewiring probability. The integration time is long enough to allow for more reliable dynamical complexity results. *Axonal conductance delays*, refer to the amount of time needed for an action potential to reach from its initiation site near the neuronal soma to the axon terminals so that it can be transmitted to other neurons through synapse connections. Axonal conductance delay between different types of neuron connections were defined as follows:

1. Excitatory-to-Excitatory (EE): $N_{\text {EE}}$ Random integers between 0 and 20 representing the delay in milliseconds are generated which are then multiplied by a scale factor of 17 to generate the axonal delays. The conduction delays vary between 0 and 20 ms.
2. Excitatory-to-Inhibitory (EI): $N_{\text {EI}}$ Random Uniform numbers between 0 and 1 representing the delay in milliseconds are generated which are then multiplied by a scale factor of 50 to produce the axonal delays. The conduction delays are all approximately 1 ms.
3. Inhibitory-to-Excitatory (IE): $N_{\text {EI}}$ Random Uniform numbers between − 1 and 0 representing the delay in milliseconds are generated which are then multiplied by a scale factor of 2 to produce the axonal delays. The conduction delays are all approximately 1 ms.
4. Inhibitory-to-Inhibitory (II): $N_{\text {II}}$ Random integers are generated between − 1 and 0 representing the delay in milliseconds which are then multiplied by a scale factor of 1 to generate the axonal delays. The conduction delays are all approximately 1 ms.

The dynamical complexity and the Mean Firing Rate (MFR) of each module for various rewiring probabilities were also computed. The dynamical complexity requires the MFR for each module in the network. The MFR is computed as described in the following steps.

1. Define a *window size* which is *greater than or equal* to the *maximum axonal conductance delay* in milliseconds (in this case the window size was set to *50 ms* because the maximum conductance delay was defined to be 20 ms).
2. Define a *slide* period in terms of milliseconds. The sliding period defines the total number of milliseconds the window defined in step 1 will be shifted. In other words, it defines the sampling rate at which the MFR is estimated using a non-overlapping window. The slide period was defined to be *20 ms*. Subsequently, using the firings that were recorded during the time of integrating the Izhikevich equations, the MFR is computed.
3. Steps 1 and 2 were carried out for **each** module that exists in the topology.

During the *Training Phase* an optimal weights matrix is derived using the Recursive Least Squares (RLS) procedure every two timesteps (each timestep of time duration $\texttt {dt}$) of the process, which is the lowest possible as it has to be computed as frequently as possible. In essence, a non-overlapping window of size two timesteps is used for this purpose. During the *Replay-State-Space Trajectory Phase*, the initial conditions of the SRNN are set to the conditions that they were at the beginning of the training phase, and the optimal weight matrix for each window derived during the Training Phase is now used without any optimization taking place.

In addition to the output a stimulus (in the form of *pink noise*, which is commonly found in neuronal activity) is also injected into the neurons in the form of ionic current during the Training Phase. This is done so that the targeted activity that has been optimized (i.e. to match the EEG signal) is ‘memorized’ and hence, is able to reproduce it when the same stimulus is injected to them during the Replay State-Space Trajectory Phase. In the Replay State-Space Trajectory Phase the Complex System is freely integrated without any Optimization taking place.

This is supported by the Synaptic Theory^44,45^ which states that stimuli induced to neurons are coded into short-term memory through the use of transmitter depletion.

### Spiking neuron model

The parameter set of the neuron voltage evolution dynamics are described as:

11 $$\begin{aligned} \frac{dv}{dt}&= 0.04v^2 +5v + 140 -u + S, \end{aligned}$$

12 $$\begin{aligned} \frac{du}{dt}&= a(bv-u), \end{aligned}$$

with the auxiliary after-spike resetting

13 $$\begin{aligned} \text {if } v \ge 30 \text {, then } {\left\{ \begin{array}{ll} v \leftarrow c,\\ u \leftarrow u + d. \end{array}\right. } \end{aligned}$$

*The parameter set for each* ***Excitatory neuron*** *is* ($\mathscr {U}$ *is a uniformly distributed random variable*):

- $a = 0.02$ is the recovery time constant (ms$^{-1}$);
- $b = 0.2$ is a constant equal to the inverse of the resistance ($10^{-9} \Omega ^{-1}$);
- $c = -65+15 \times (\mathscr {U}(0,1))^{2}$ is the potential after-spike reset value (mV);
- $d = 8-6 \times (\mathscr {U}(0,1))^{2}$ is the outward minus the inward ionic currents which are activated during the spike and consequently affecting the after-spike behavior (*pA*).
- usage of double exponential synapse type (see section “Synapse types”)

*The parameter set for each* ***Inhibitory neuron*** *is as follows* ($\mathscr {U}$ *is a uniformly distributed random variable*):

- $a = 0.02+0.08 \times (\mathscr {U}(0,1))^{2}$ is the recovery time constant (ms$^{-1}$);
- $b = 0.25-0.05 \times (\mathscr {U}(0,1))^{2}$ is a constant equal to the inverse of the Resistance ($10^{-9} \Omega ^{-1}$);
- $c = -65$ is the potential after-spike reset value (*mV*);
- $d = 2$ is the outward minus the inward ionic currents which are activated during the spike and consequently affecting the after-spike behavior (*pA*).

The topology is set to be small-world modular and various rewiring probabilities are examined with the performance metrics to be used being RMSE and dynamical complexity.

Full-Force fundamentally uses feedback loops to reorganise *chaotic behaviour* to generate complex, but controlled outputs. Let’s formally define the key parts of the full-Force algorithm and subsequently define the pseudocode. First, a $J \in \mathscr {R}^{N \times N}$ adjacency matrix which portrays the connectivity and synaptic strengths between neurons in the reservoir is required. The matrix contains both excitatory and inhibitory synapses. *Dale’s law* states that a neuron *cannot* be both excitatory and inhibitatory. These are *hard constraints* for the connectivity matrix configuration and throughout the learning process. The activity of the reservoir is computed using Numerical Approximation methods of a neuron model (e.g. the Izhikevich coupled first order differential equations over the connectivity matrix *J* which defines the synaptic strengths between neurons). At the time instant, *t*, the neuronal activity is denoted as $\mathbf{r} (t) \in \mathscr {R}^{N}$. In general, $\mathbf{r} \in \mathscr {R}^{N \times T}$ where *T* denotes the whole time horizon of integration. The output of the reservoir, *z*(*t*), is a scalar value which is formed by the synapse strength between the neurons denoted as $\phi (t) \in \mathscr {R}^{N}$:

14 $$\begin{aligned} y(t) \approx z(t) = \phi (t)^{T}\varvec{r}(t) \end{aligned}$$

It should be noted that $\mathbf{W} _{out}$ is now denoted as $\phi (t)$. In general, $\mathbf{z} \in \mathscr {R}^{T}$, where *T* is the total integration time across the time horizon (i.e. $\mathbf{z}$ is a sequence of spatio-temporal scalar values). The target signal that needs to be approximated is denoted as $\mathbf{y} (t) \in \mathscr {R}^{T}$. In other words, the following *Optimization problem* needs to be solved:

15 $$\begin{aligned} \min _{0 \le t \le T} \left\{ \int _{0}^{T} (\text {z}_{task}\text {(t) - uz}_{target}\text {(t) - y(t)})^{2} \text {dt} + \alpha \phi (t)^T \phi (t) \right\} \end{aligned}$$

where $\alpha >0$, ‘u’ is dimensionless and sampled from a uniform distribution in the interval − 1 and 1, $\phi (t)$ is the learned readout optimal weights layer at each iteration step and $\alpha \phi (t)^T \phi (t)$ is the Tikhonov regularization matrix. Therefore, the optimal weights can be formed as:

16 $$\begin{aligned} \varvec{\phi (t)} = \mathbf{r} ^{-1}{} \mathbf{z} \end{aligned}$$

It is possible that the matrix $\mathbf{r}$ is not invertible, because it may not be a full-rank matrix and hence no unique solution may exist. Thus, in order to solve this problem, a *Recursive Least Squares* (RLS) adaptive filter can be used for obtaining an approximation of the solution. *x*(*n*) denotes the input signal at time point *n* (i.e. in this case the neuron stimulus), $w_n$ denotes the weights to be learned, $\hat{d}(n)$ denotes the approximated output, *d*(*n*) denotes the target output and finally, *e*(*n*) denotes the error between the estimated output and the target output which is fed back to the update algorithm (i.e. RLS). More on how RLS is integrated into the learning procedure is described later on in this section.

### Discussion

The main goal of this work is to propose a new and complete framework that: (a) is more biologically plausible than the current state-of-the-art, but still computationally efficient as a trade-off; and (b) can generate time-series signals which can closely match real-life brain signals, such as EEG.

The connectivity of the neural network (i.e. the topology), plays a vital role in the neural dynamics of the human brain. Hagmann et al.^33^ parcellated the cortical surface on the brain (i.e. cerebral cortex) into regions which represented the nodes of the network. There exists an edge between any two nodes where White Matter data shows a fibre tract. Subsequently, the edge weight is set according to the thickness of the fibre track. Therefore, their results showed that the brain is a modular SWN. In addition, Riecke et al.^15^ indicated that the topology generated by the Watts–Strogatz model can be considered approximately equal to a medium sized area of the cortex. The modular SWN topology is also supported by the Global Workspace Theory^46^, which is one of the most well-known theories of consciousness. It should be noted that SWNs can exhibit short-term memory, which is the ability to hold information between different states in a dynamical system.

In the work done in this paper. Several contributions have been made towards unlocking the secrets of the brain by constructing a novel CBM.

The first direct application of the full-Force method to a spiking neuron model with conductance delays has been successfully demonstrated, experimentally proving the claims in^3^ regarding its agnostic nature for the network topology. Also, the first direct applicability of the full-Force method to modular SWNs was demonstrated in order to explicitly enforce biological plausibility to the CBM. Results showed that it was indeed enforced, given the MFR patterns and the relatively high value of SWI computed after the topology for each network in the experiments was initialized.

Moreover, the aforementioned method, applied to spiking neuron models, is trained on various real-life EEG signals for the first time. Also, a CBM is proposed for the first time which is a trade-off between high biological plausibility, measured by the Information Theoretic metric of dynamical complexity (which resembles the Consciousness of the brain) and low regression error between the target and the modelled EEG signal, measured by RMSE. The same method is applied to both a fully undirected and a fully directed network. It is evident from the results that directionality in synapse connections vastly affects both of the two metrics, since in the former, both the dynamical complexity and the RMSE are higher than the latter, thus highlighting the trade-off that exists between the two metrics. All experiments have been repeated on the 32 EEG channels of the EEG dataset in order to explore the spatiotemporal dynamics of the brain. Results have shown that both categories of networks performed well, exhibiting similar patterns in terms of RMSE.

Additionally, a new method termed *modified-full-Force* has been proposed by modifying the original full-Force method. Thus, making it a learning rule which is more biologically-plausible and also less computationally expensive. In contrast to the original one, the newly proposed algorithm does not makes any modifications through feedback to the neurons in the network topology and does not as a result tune the whole neural network’s population to the same spatiotemporal dynamics which is biologically implausible. If such a feedback term was still enforced, the Dynamical Complexity of the neural network would no longer be maximized as is the case in this paper. Having a feedback term is also more computationally expensive as all the neurons in the network have to be updated at the end of each iteration of the algorithm. A new MATLAB toolbox was developed and released open-source that can take any kind of time-series signals (e.g. EEG) and reproduce all the results of this paper. The specific EEG dataset in this paper can be found at^47^.

#### Methods

### EEG modelling

#### EEG dataset

The EEG dataset used is composed of (already processed) continuous EEG data collected during a selective visual attention experiment^47^. The format of the original dataset is a $32 \times 384 \times 80$ spatiotemporal multivariate time-series, representing the 32 channels of the EEG, the 384 data points (in time) and 80 epochs. 32 (single-channel) time-series signals of 384 data points each. The data were then augmented, as described in the next section.

#### Data augmentation

Using interpolation, the original data is used to expand its original size. In the context of neuron models, the introduction of more datapoints allows for a numerical approximation with *greater precision*.

The datapoints in each of the 32 EEG time-series signals were increased using a method known as *Lowpass Interpolation*^48^ in Signal Processing which is the opposite of decimation. This was accomplished by increasing the original sampling rate, $f_1$, of the spatiotemporal EEG sequence into a higher one $f_2 > f_1$. Using a closed-loop feedback the error is propagated for adjustment of the weights matrix used in the Recursive Least Squares (RLS) adaptive filter during full-Force learning.

Denote the original EEG signal to be interpolated as $y_1 \in \mathscr {R}^{T_1}$ and the interpolating signal as $y_2 \in \mathscr {R}^{T_2}$, where $T_2>> T_1$. The algorithm applied is the following:

1. Zero-pad the original signal, of length $T_1$, between the locations of the original data points in order to increase its length from $T_1$ to $T_2$.
2. A symmetric Finite Impulse Response (FIR) filter which allows the original data to pass through unchanged is used. Additionally, the FIR filter generates new datapoints with the objective of minimizing the Mean Squared Error (MSE) between the original data points and the interpolated ones. This is achieved through an ideal bandlimited interpolation using the nearest 2*p* (where $p > 0$) non-zero samples, on a sequence interleaved with $l-1$ consecutive zeros every *l* samples.

Through this process, the EEG signals have been brought into a *higher-dimensional* space. This allows the use of smaller integration step sizes that result in better approximation and hence, capturing more precise details of the signal’s dynamics.

### Synapse types

From the neuronal spikes that have fired, a filter is applied according to the specific synapse type. There are three filter types that can be used.

1. Simple exponential synapse
2. Double exponential synapse
3. Alpha synapse

For single exponential synaptic filters (i.e. when the synaptic decay time for a neuron is 0 ms), the neuronal activity vector is modified as:

17 $$\begin{aligned} \dot{{r}_{i}}&= \frac{-\mathbf{r} _{i}}{\tau _{s}}+\frac{1}{\tau _{s}\tau _{d}}\sum _{t_{ik}<t} \delta (t-t_{ik}) \end{aligned}$$

where $\tau _s$ is the synaptic time constant for the filter and $t_{ik}$ represents the $k^{th}$ spike fired by the $i^{th}$ neuron. On the other hand, the double exponential filter (i.e. when the synaptic decay time is greater than 0 ms) is given by:

18 $$\begin{aligned} \dot{{r}_{i}}&= \frac{-\mathbf{r} _{i}}{\tau _{d}} + \mathbf{h} _{i} \end{aligned}$$

19 $$\begin{aligned} \dot{{h}_{i}}&= \frac{-\mathbf{h} _{i}}{\tau _{r}}+\frac{1}{\tau _{r}\tau _{d}}\sum _{t_{ik}<t} \delta (t-t_{ik}) \end{aligned}$$

where $\tau _{r}$ represents the synaptic rise time, $\tau _d$ represents the synaptic decay time. Finally, for an alpha synapse type, $\tau _d = \tau _r$. Synaptic currents in the the networks are given by:

20 $$\begin{aligned} z_i = \sum _{i=1}^{N} \phi (t)_{ji}r_{i} \end{aligned}$$

where $\phi (t)_{ji}$ denotes the adjacency matrix of synaptic connections in each network and hence, controls the absolute value of the postsynaptic currents that arrive from neuron *i* to neuron *j*.

The objective of the training procedure is to achieve a good estimation of the exhibited dynamics modelled by a reference target signal (i.e. $\mathbf{z} \approx \mathbf{y} \in \mathscr {R}^{M}$). **z** is derived from $\phi (t)^{T}{} \mathbf{r}$, where $\phi (t)$ is the linear decoder in regards to the firing rate of the neurons in the task-generating network. The full-Force algorithm breaks down the static weight matrix of each network, ${\varvec{\phi (t)}}$ to:

21 $$\begin{aligned} \xi _{ji} = G {\xi }_{ji}^{0}+Q \varvec{\eta }_j \phi (t)^{T} \end{aligned}$$

The matrix ${\xi }_{ji}^{0}$ represents the static weights which produce the chaos in the dynamical system. Its entries are sampled from a normal distribution with 0 mean and $\frac{1}{Np}$ variance, where *p* defines the randomness of the Watts–Strogatz model. The *G* parameter defines the chaos behaviour within the network. The parameter $\eta _j$ represents a set of samples drawn from a uniform distribution in the range − 1 to 1. Its dimension matches the dimension of the target signal. Finally, the parameter *Q* controls the dynamics of the recurrent connections when chaos is induced within the network.

### Full-FORCE equations

The pseudocode of the full-Force algorithm is provided in Algorithm 2 followed by a detailed analysis of each step in the process. Note that the ${\varvec{\xi }}$ letter is used for the static initial adjacency matrix of the TASK network and $\nu$ for the TARGET network. Moreover, the following pseudocode assumes that the target signal to be approximated is 1-Dimensional.

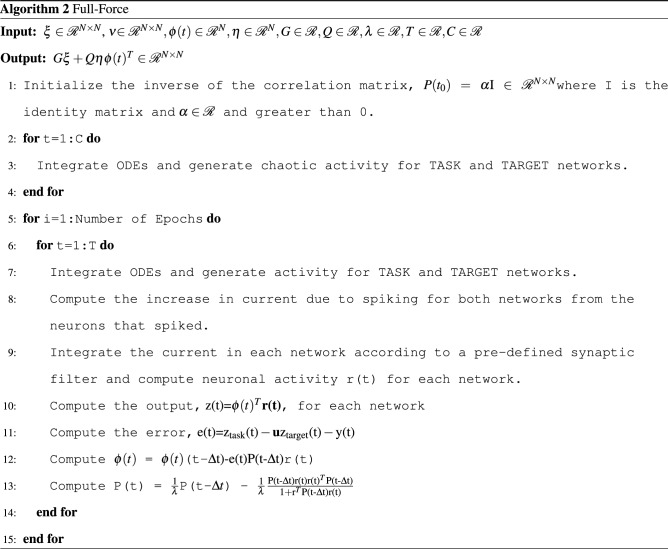



First, the two networks are instantiated. These networks are initialized in the form of $N \times N$ adjacency matrices. In terms of inputs, the time horizon during which modelling takes place is defined. Subsequently, the chaos period needs to be defined so that the networks can get to a state in which they can resemble properties that are exhibited by *Nonlinear Dynamical Systems*. In STEP 1, the inverse of the correlation matrix needs to be initialized. The correlation matrix *P*(*t*) represents the correlation between neuronal activities in the network. Its closed form expression is given by:

22 $$\begin{aligned} P(t)^{-1} = \int _{0}^{T} \mathbf{r} (t)\mathbf{r} (t)^{T} dt + \alpha I \end{aligned}$$

where $I \in \mathscr {R}^{N \times N}$ is the identity matrix and $\alpha$ is a regularization parameter which is strictly positive. As previously mentioned, $\mathbf{r} (t)$ indicates neuronal activity. Since no neuronal activity has yet to be initialized through Numerical Approximation of the respective ODEs that describe the neuron model dynamics, the term $\mathbf{r} (t)\mathbf{r} (t)^{T} = 0$. In STEPS 2–4, the networks are brought into a chaotic regime for the reasons previously mentioned. In STEPS 5–13, the neuron models are solved for using a defined numerical approximation method. The parameter $\lambda$ in STEP 15 is the forgetting factor in the RLS adaptive filter. The forgetting factor can take values $0 \le \lambda \le 1$ and it defines how much past data will still be considered during optimization. If $\lambda = 1$, then the RLS is in its *growing window* form, where all the previous errors are taken into account for computation of the optimal weights at the current optimization step. The process can be repeated for a given number of epochs in order to allow the weights to converge from (incorrect) initial conditions.

## Supplementary Information

Supplementary Information.

## Data Availability

The code and data used for this paper can be found on GitHub^49^.
