# Supplementary material for: Spatiotemporal dynamics in spiking recurrent neural networks using modified-full-FORCE on EEG signals
Source: Sci Rep. 2022 Feb 21;12:2896. doi: 10.1038/s41598-022-06573-1 (PMC8861015; doi:10.1038/s41598-022-06573-1)
Supplement: Supplementary file 1 — Supplementary Information. [file 41598_2022_6573_MOESM1_ESM.pdf]

Supplemetary Material

Results

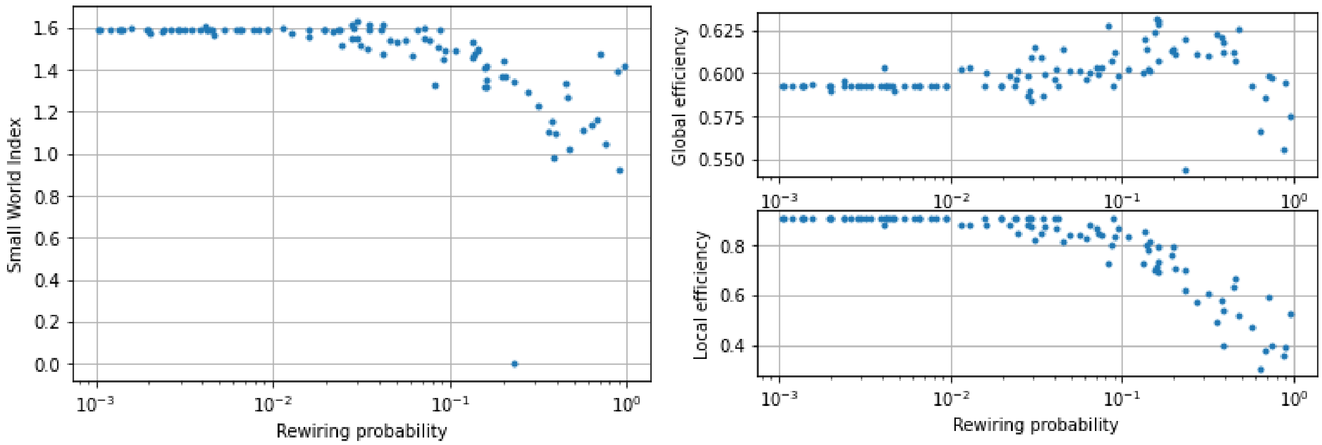

Figure S1. Graph Theoretic metrics for 20 nodes in a modular SWN. Blue dots show variation of SWI with rewiring probability (left). Blue dots show variation of GE (top) and LE (bottom) with rewiring probability (right).

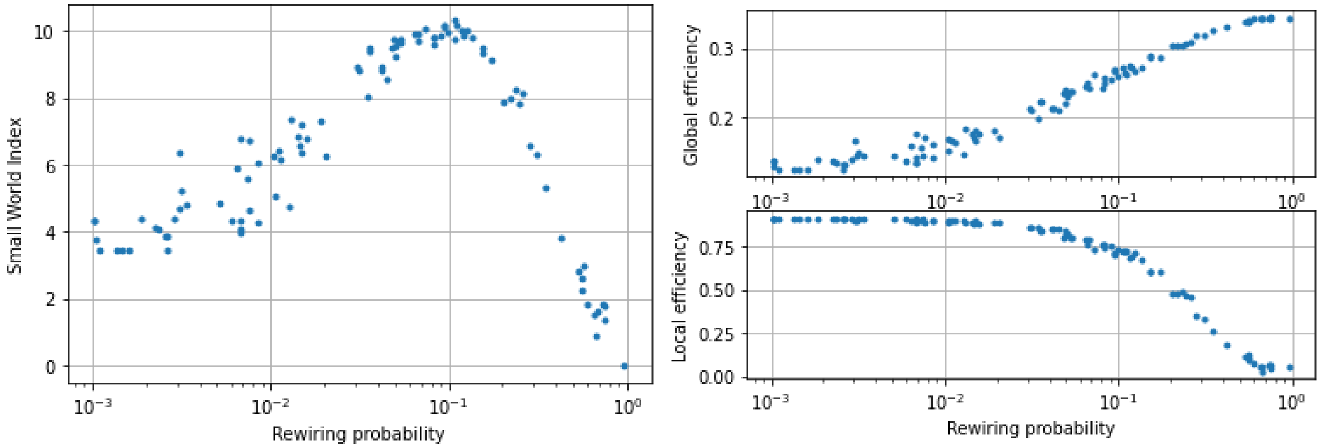

Figure S2. Graph Theoretic metrics for 200 nodes in a modular SWN. Blue dots show variation of SWI with rewiring probability (left). Blue dots show variation of GE (top) and LE (bottom) with rewiring probability (right).

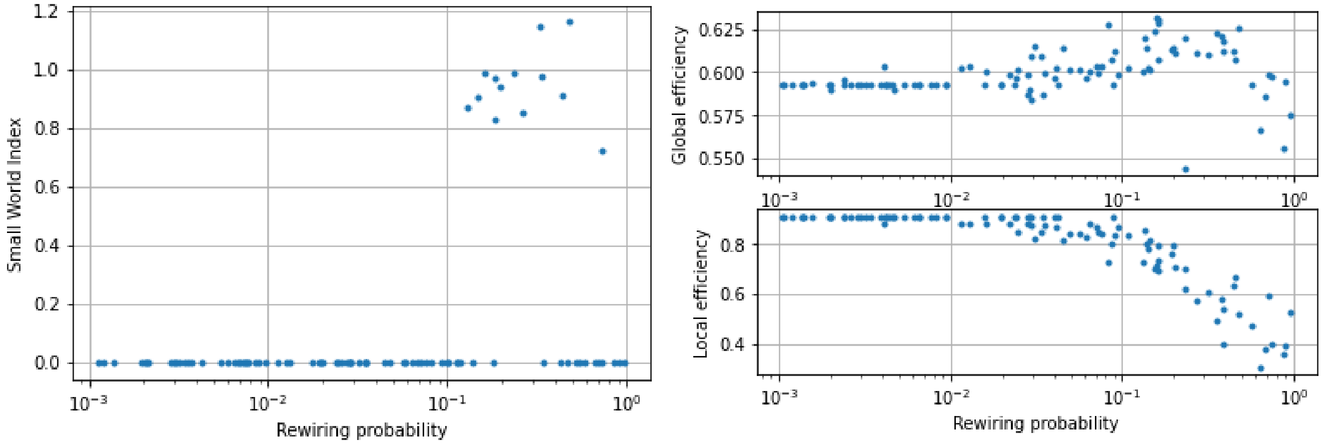

Figure S3. Varying rewiring probability for 20 nodes in an NSN. Blue dots show variation of SWI with rewiring probability (left). Blue dots show variation of GE (top) and LE (bottom) with rewiring probability (right).

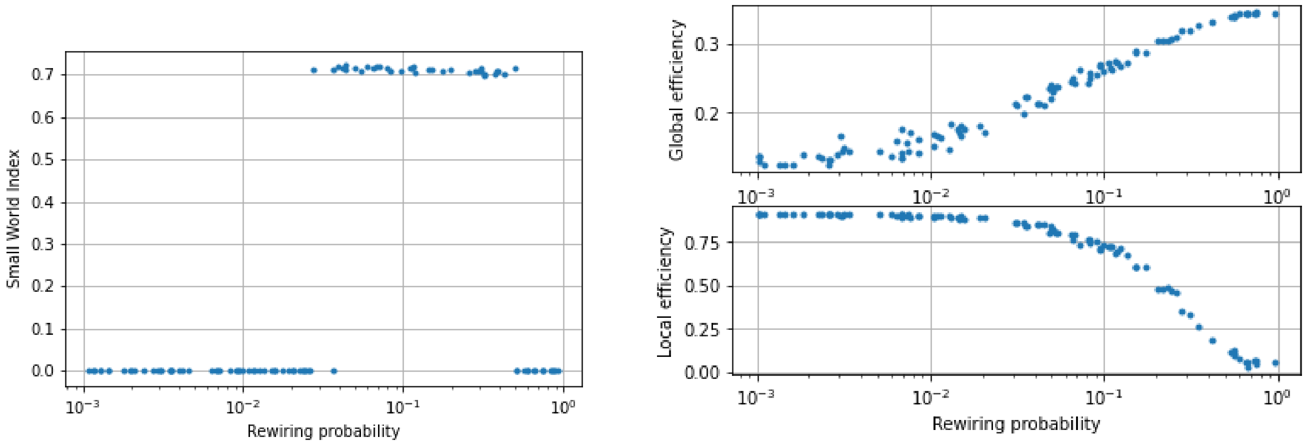

Figure S4. Varying rewiring probability for 200 nodes. Blue dots show variation of SWI with rewiring probability (left). Blue dots show variation of GE (top) and LE (bottom) with rewiring probability (right).

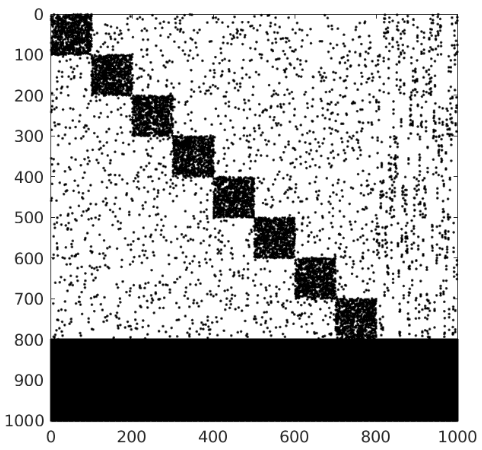

Figure S5. Rewiring probability of 0.2. Black dots show connection between respective neuron indexes.

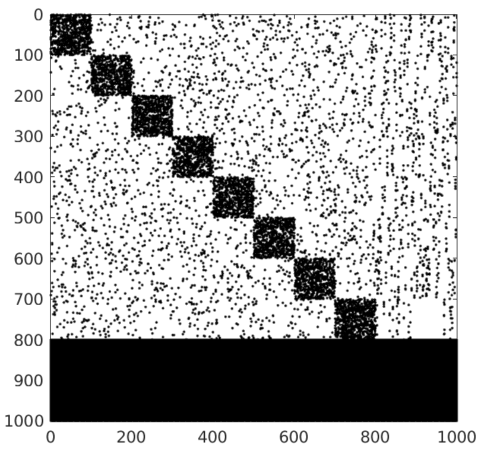

Figure S6. Rewiring probability of 0.3. Black dots show connection between respective neuron indexes.

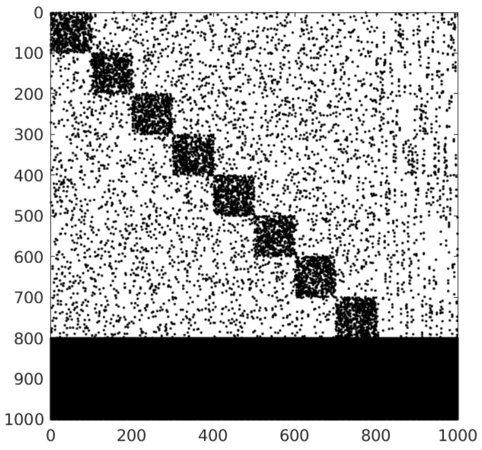

Figure S7. Rewiring probability of 0.4. Black dots show connection between respective neuron indexes.

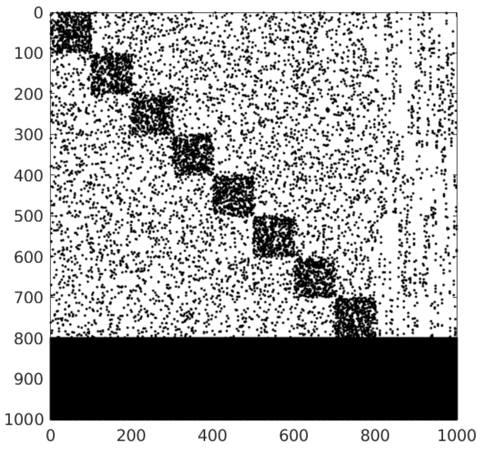

Figure S8. Rewiring probability of 0.5. Black dots show connection between respective neuron indexes.

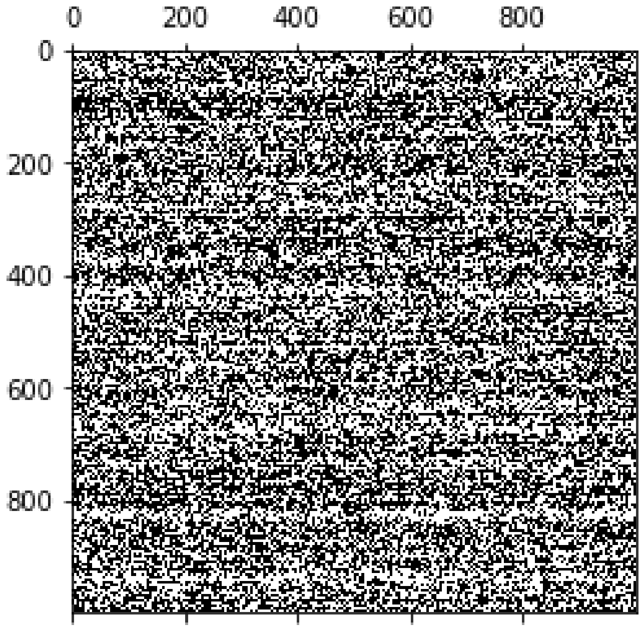

Figure S9. Naive Sparse Network w/ Rewiring probability of 0.1. Black dots show connection between respective neuron indexes.

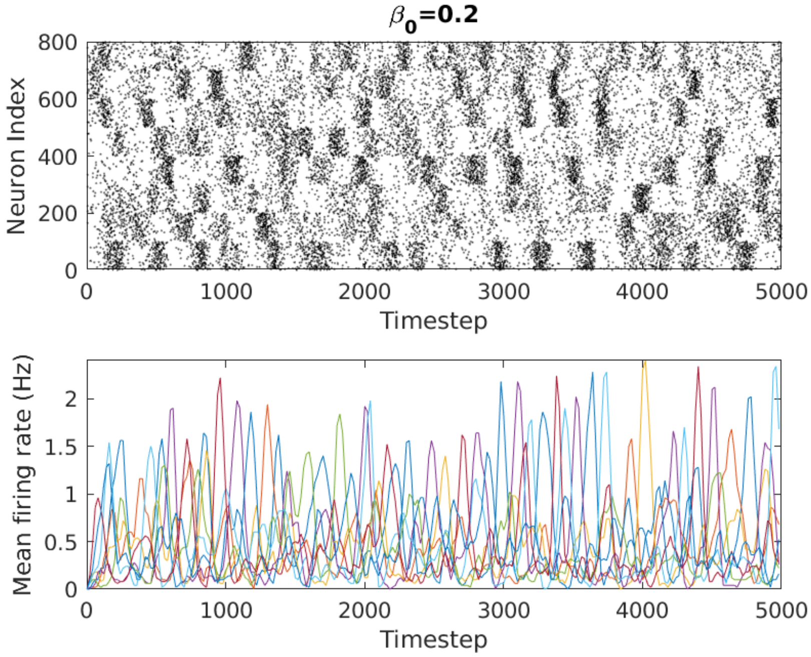

Figure S10. Rewiring probabilities of 0.2 (raster plot).

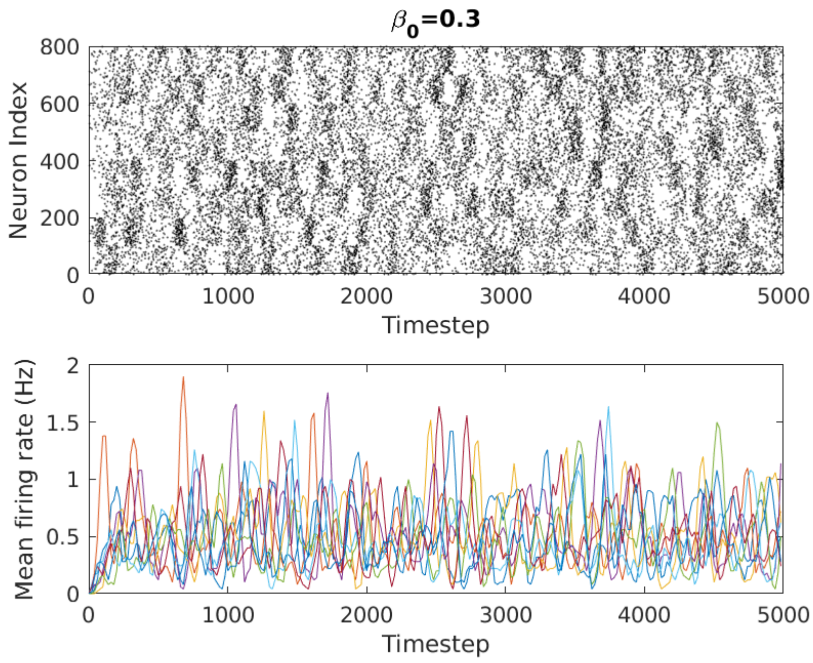

Figure S11. Rewiring probabilities of 0.3 (raster plot).

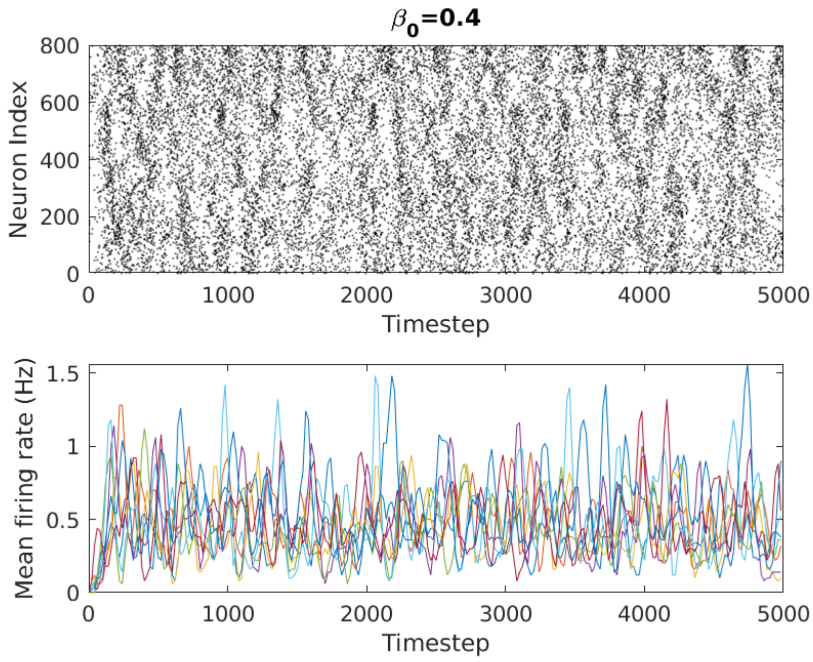

Figure S12. Rewiring probabilities of 0.4 (raster plot).

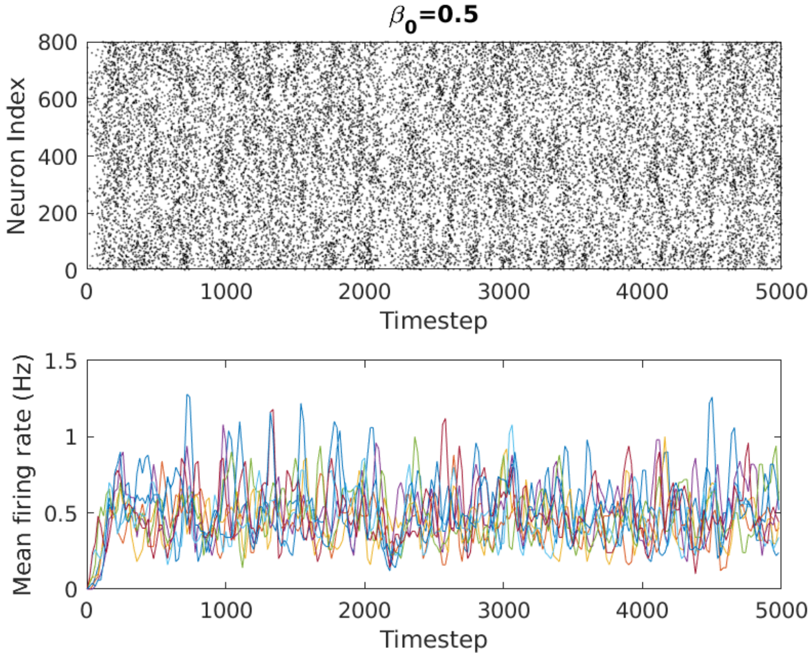

Figure S13. Rewiring probabilities of 0.5 (raster plot).

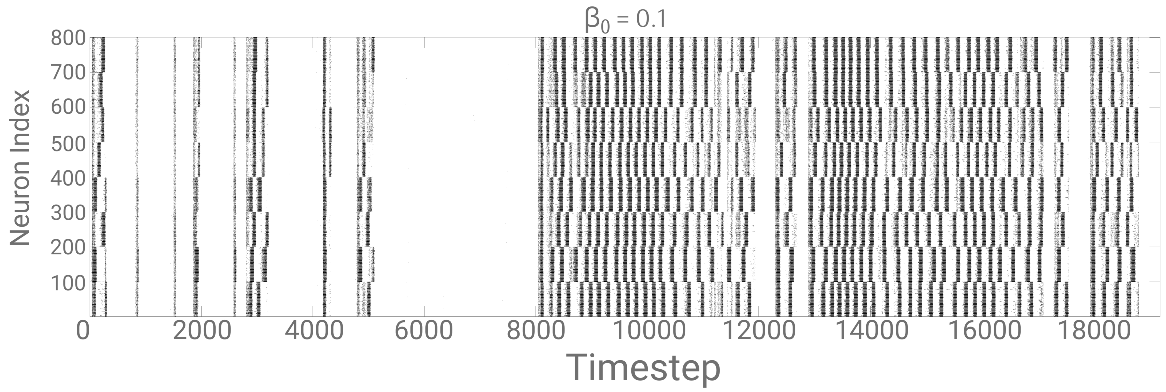

Figure S14. Rewiring probabilities of 0.1 using the original full-FORCE method (raster plot).

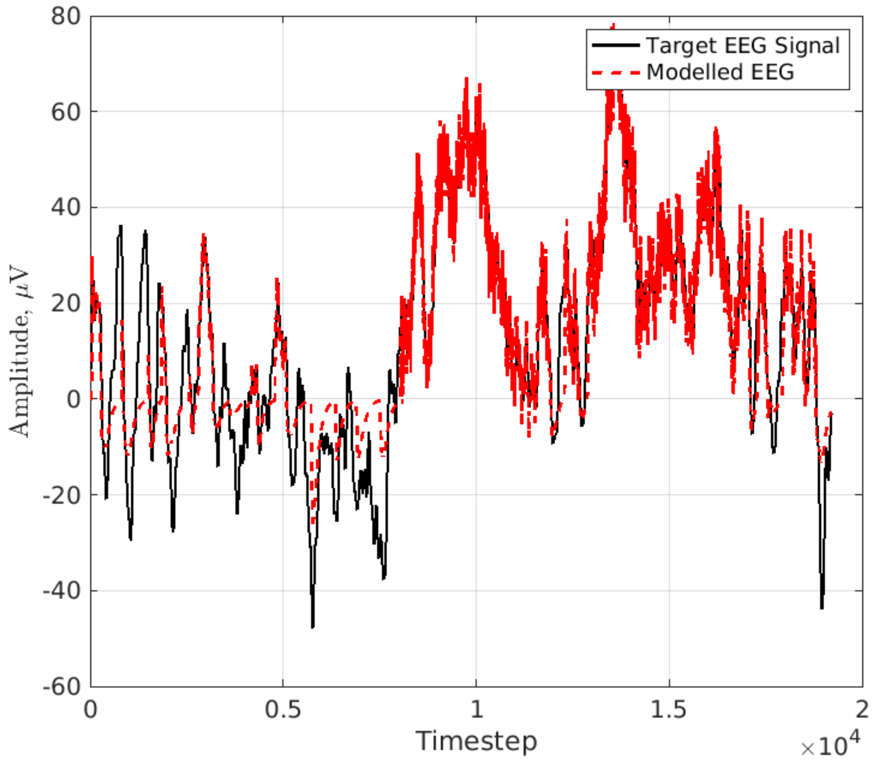

Figure S15. Modelled EEG signal using the original full-FORCE method (channel 14).
